# Special Issue on Mechanisms of Mesothelioma Heterogeneity: Highlights and Open Questions

**DOI:** 10.3390/ijms19113560

**Published:** 2018-11-12

**Authors:** Emanuela Felley-Bosco

**Affiliations:** Laboratory of Molecular Oncology, University Hospital Zurich, Sternwartstrasse 14, 8091 Zürich, Switzerland; emanuela.felley-bosco@usz.ch

**Keywords:** mesothelioma heterogeneity, NF2/Hippo pathway, BAP1, non-coding RNA, tumor microenvironment, experimental models

## Abstract

This editorial aims to synthesize the eleven papers that have contributed to this special issue, where the mechanisms of mesothelioma heterogeneity have been tackled from different angles.

A general feature of a tumor is that it comprises tumor cells and stroma containing immune cells, fibroblasts, matrix and blood vessels. Therefore, it is not surprising that in this special issue, the mechanisms of mesothelioma heterogeneity have been addressed extensively at the level of tumoral cells, highlighting differences in genetic alterations [1,2,3,4,5] or temporal differences during tumor progression [2]. 

In this context, it is worth noting that besides the two pathways widely mutated in cancer, namely, cell cycle control (cyclin-dependent kinase Inhibitor 2A, *CDKN2A*) and genome integrity (*TP53*), there are also two specific pathways frequently mutated in MPM, namely, the neurofibromatosis type 2 (*NF2*)/Hippo and the Breast-Repair-associated-Cancer 1(BRCA)-associated protein 1 (*BAP1*) pathways. 

With regard to NF2/Hippo, as pointed out by Sato and Sekido [5], it is intriguing that if their downstream targets are activated yes-associated protein 1 (YAP1) and transcriptional co-activator with PDZ domain-binding motif (TAZ), no mutations that result in their activation have been observed in mesothelioma. Mutations that result in their constitutive activation would involve mutations of individual or multiple phosphorylation sites, allowing YAP and TAZ retention in the cytosol preventing activation of YAP/TAZ-dependent transcription. However, there are well-known examples, like Phosphatase and tensin homolog (PTEN), where loss of control of phosphorylation targets are tumorigenic. In addition, both YAP and TAZ have multiple phosphorylation sites so it is likely that deregulation of the upstream kinase would be more efficient. As reviewed by Sato and Sekido [5], YAP has been largely investigated in mesothelioma, however, Hagenbeeck et al. [6] recently noted that YAP and TAZ have slightly different transcriptional profiles, whereby TAZ increases, for example, the expression of wound-healing-associated, pro-tumorigenic genes such as *Arginase 1*. This gene was one of the genes with the highest expression in tissues from asbestos exposed mice and remained high in tumors [7]. Therefore, there remains an open question about a possibly synergistic mode of action where TAZ modifies the tumor microenvironment while YAP promotes tumor cell proliferation.

While the understanding of the mechanisms behind the contribution of the NF2/Hippo pathway to mesothelioma has progressed greatly since the seminal observation of the high frequency of *NF2* mutations in mesothelioma [8,9], understanding of the mechanisms underlying BAP1 are less advanced. This is to be expected as this mutational event was discovered more recently [10,11]. Interestingly, in the analysis of TCGA samples, *BAP1* status was associated with differential gene expression [12] as originally described in Drosophila (fruit fly). Here the BAP1 homolog was responsible for repression of *HOX* genes in the fly embryo while also increasing *HOX* expression in particular tissues in central nervous system [13].

Because of the known role of long non-coding RNA (lncRNA) in assembling and controlling transcriptional complexes (reviewed in [14]), it would be of interest to explore if lncRNA associated with BAP1 show differential transcriptional profiles that are associated with better clinical outcome [12,15]. In fact, their expression may, for example, point to a given cell of origin and commitment to epithelial differentiation phenotype. This was observed in patients’ samples by Felley-Bosco and Rehrauer [16] for *FENDRR*, a lncRNA found to be overexpressed in tumors developing in mice after exposure to asbestos fibers, and which also clusters with better outcomes in human mesothelioma patients [12]. Similarly, *Meg3*, another lncRNA found to be overexpressed in tumors developing in mice after exposure to asbestos fibers [16] is overexpressed in TCGA cluster 1, which was characterized by better overall survival [12] compared to the other 3 clusters of patients with different transcription profiles. 

Other non-coding RNA of interest that have been extensively reviewed [17] include microRNA (miR), which have been deeply investigated for diagnostic and prognostic purposes and reviewed by Martinez-Rivera et al. [17]. They highlight the challenges to come with the investigation of circulating miR in total plasma/serum vs exosomal vesicles. In this context, additional complexity has been recently added by the investigation of expression obtained through RNA-seq data. This has revealed how classical analysis approaches may miss isomiRs [18].

Even though peritoneal mesothelioma is less frequent compared to pleural mesothelioma, the mutational landscape is similar, with *BAP1* frequently being mutated [19]. The reported case of long-survivor peritoneal mesothelioma by Serio et al. [4] did not display any of the mutations in the frequently mutated genes *BAP1*, *CDKN2A*, or *NF2* and was treated with oxaliplatin, a known inducer of immunogenic cell death [20]. Therefore, if more tissue were available from mesothelioma patients treated with oxaliplatin, it would be interesting to establish a cohort where potential neoantigens generation and immune response could be explored.

Heterogeneity in the tumor environment has been widely reviewed [1,21] with more emphasis on heterogeneity in immune cell content in the tumor microenvironment, which is also in line with the intensive exploration of immunotherapy in mesothelioma treatment [22]. Minnema-Luiting and colleagues [21] emphasize how several studies point to the important role of M2-polarized macrophages in mesothelioma. Interestingly, according to the interactive web-based platform https://www.cri-iatlas.org/ [23], which was established as an analytic tool for studying the interactions between tumors analyzed in TCGA and the immune microenvironment, the best relationship with leukocytes tumoral infiltration is observed for the signature known as the “macrophage regulation” (Figure 1a) This is better when compared to the relationship with the signature called the “IFN-gamma response” (Figure 1b). Altogether, these observations point to the macrophage population as a major regulator of the immune system in mesothelioma.

Besides immune cells, the mesothelioma tumor environment also contains cancer-associated fibroblasts and a matrix, likely produced by the tumor, immune cells and fibroblasts themselves. However, both cancer-associated fibroblasts and the matrix, which are likely to be major contributors of stiffness-dependent effects such as modulation of YAP/TAZ transcriptional regulators [24], remain to be explored.

As highlighted by Tolani et al. [1], a stem cell signaling pathway that should be further explored is the Notch signaling pathway, especially since it is expressed in patients with predominantly non-epithelioid histologies with poorer outcomes [12] compared to patients in cluster 1, who are characterized by better overall survival.

Jean and Jaurand wrote a timely, comprehensive review on how experimental murine mesothelioma models [25] have helped in understanding the mechanism of mesothelioma development using tissue specific targeted gene disruption using injections of AdenoCre or exposure to asbestos fibers. Genetic alteration signatures observed in mice exposed to asbestos resemble what is observed in human clinical samples and is mostly associated with copy number variations. This is in line with the lack of detection of a specific point mutation signature (https://cancer.sanger.ac.uk/cosmic/signatures), besides aging, in the two-human high-through-put studies [12,26]. These models are useful for the investigation of other relevant changes, such as epigenetic modifications.

Finally, yet importantly, Colin et al. [27] developed a human orthotopic (intrapleural) xenograft model in athymic mice, where it is possible to investigate the role of macrophage migration inhibiting factor (MIF) because this particular model expresses both MIF and its functional receptor CD74. The authors show the presence of M2-polarized macrophages in this model. Therefore, the model allows not only investigating the role of MIF but also testing drugs acting on macrophage polarization, thus allowing testing of the effect of macrophage polarization on tumor growth. 

## Figures and Tables

**Figure 1:**
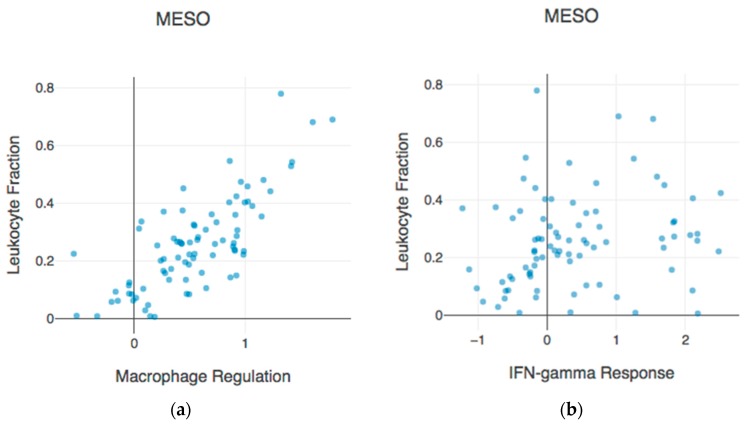
Mesothelioma leukocyte fraction is highly correlated with the signature “Macrophage regulation” (**a**) compared with the correlation with IFN-gamma response (**b**). These graphics were obtained using the interactive web-based platform https://www.cri-iatlas.org/ [23].
